# Editorial: Comorbidities in Women With Endometriosis: Risks and Implications

**DOI:** 10.3389/frph.2022.875277

**Published:** 2022-06-27

**Authors:** Sarah J. Holdsworth-Carson, Cecilia H. M. Ng, Uri P. Dior

**Affiliations:** ^1^Department of Obstetrics and Gynaecology, University of Melbourne and Gynaecology Research Centre, Royal Women's Hospital, Parkville, VIC, Australia; ^2^Julia Argyrou Endometriosis Centre, Epworth HealthCare, Richmond, VIC, Australia; ^3^Jean Hailes for Women's Health, East Melbourne, VIC, Australia; ^4^School of Clinical Medicine, University of New South Wales, Sydney, NSW, Australia; ^5^Global Women's Health Program, The George Institute for Global Health, Sydney, NSW, Australia; ^6^Department of Obstetrics and Gynecology, Endometriosis Center, Hadassah-Hebrew University of Jerusalem, Jerusalem, Israel

**Keywords:** endometriosis, comorbidities, endometrium, pain, fibroids

Women with endometriosis suffer lengthy diagnostic delay. In part, this is the consequence of nonspecific and overlapping symptomology with common gynecological and other diseases. As such, endometriosis may be confused or misdiagnosed as an entirely different condition. The challenge for health professionals is increased further by growing observations indicating that people with endometriosis also demonstrate a high incidence of gynecological and non-gynecological comorbidities and chronic conditions. The aim of our Research Topic was to highlight existing evidence-based research, or lack thereof, that aligns comorbid and chronic conditions with endometriosis.

The Research Topic received two manuscripts describing non-gynecological comorbidities associated with endometriosis. The first, Genetic Relationship Between Endometriosis and Melanoma by Yang et al. There is a large body of literature on the possible association between endometriosis and various malignancies, including ovarian, breast, thyroid, colon and skin cancers. Yang et al., focused on melanoma and used summary statistics from genome-wide association meta-analyses to estimate if there is a genetic correlation between endometriosis and melanoma. Despite no correlation between endometriosis and a wide range of skin traits, after restricting the analysis to female cohorts, they have indeed found a significant positive correlation between melanoma and endometriosis with the identification of 27 genomic loci that are associated with the two diseases. These findings show that an increased risk for melanoma in females may be related to an increased risk of endometriosis. As the authors clearly state, further and larger studies are needed to confirm or refute those findings.

The second non-gynecological comorbidity paper received was by O'Malley et al. and reviewed evidence for an association between endometriosis and allergic and non-allergic food hypersensitivities. There is substantial crossover between symptoms associated with food hypersensitivities and endometriosis-related gastrointestinal symptoms, including abdominal/pelvic pain, dyschezia, bloating, nausea, vomiting, flatulence, and diarrhea, yet evidence supporting a relationship between the two is limited. Therefore, O'Malley et al. carried out a quasi-systematic review to explore research advances which associated food hypersensitivities with endometriosis. The authors uncovered two major concerns. Firstly, the dogma that patients with endometriosis experience food allergy and/or intolerance at a greater frequency than women without endometriosis is derived from a singular small study, undertaken almost 40 years ago, that reported minimal statistical significance and omitted a description of how endometriosis diagnoses were made. Secondly, they surmise that the misinformation linking food hypersensitivities and endometriosis has not been corrected because there is a substantial lack of recent, high-quality, evidence-based research undertaken on the topic. They conclude that until investigations better explore the relationship between endometriosis and allergic and non-allergic food hypersensitivity, the true association with endometriosis will remain unknown.

Our Research Topic received three manuscripts describing gynecological comorbidities associated with endometriosis. McNamara et al. describes the Peripheral, Central, and Cross Sensitization in Endometriosis-Associated Pain (EAP) and Comorbid Pain Syndromes. Endometriosis pain management can be complicated by not only the local effects of disease, but the existence of comorbid pain syndromes such as bladder pain syndrome, irritable bowel syndrome, abdomino-pelvic myalgia and vulvodynia. In their review, McNamara et al., discuss the available literature to aid clinicians in navigating the terminology, mechanisms and commonalities between EAP and comorbid pain syndromes. In describing EAP, the authors noted different descriptions and definitions, having evolved from different clinical guidelines and classifications, and subsequently applied—either as a subset of persistent pelvic pain or as a discrete condition. However, it remains unclear whether endometriosis is coexistent or causative and is restricted by the current limitations of understanding endometriosis. Mechanisms common and contributing to both EAP and comorbid pain syndromes include peripheral, central and cross-sensitization. The authors conclude that it is imperative for clinicians to take note of any biological, psychological and/or social factors contributing to the patients' EAP to screen for comorbid pain syndromes.

Uterine fibroids (leiomyomata), like endometriosis, is a common estrogen-responsive gynecological condition that is responsible for overlapping symptoms including pelvic pain and sub-fertility. Uimari et al. reviewed the literature to summarize the Comorbidity, Risks and Implications of Endometriosis and Uterine Fibroids. They found that retrospective studies reported varied degree of comorbidity between endometriosis and fibroids, ranging from 12 to 87.1% for fibroids with comorbid endometriosis. Despite the two pathologies having distinct developmental trajectories, the authors emphasize common genetic underpinnings that recent GWAS meta-analyses have revealed that increase risk for both endometriosis and fibroids; on chromosome 1 encoding for *CDC42* and *WNT4*, chromosome 2 for GREB1, chromosome 6 for *SYNE1* and *ESR1*, and chromosome 11 for *FSHB*. Uimari et al., recommend that “given the substantial comorbidity between the conditions, it might be beneficial to factor in surgery for one condition when addressing the other, so as to avoid the need for repeating surgical procedures. (As) treatment of one condition while ignoring the other could fail to address the patient's complaint”.

Lastly, the third gynecological comorbidity paper was a review that looked at Obstetric Outcome after Surgical Treatment of Endometriosis by Mooney et al. The authors examined the evidence describing the association of adverse obstetric outcomes after 20 weeks' gestation and pre-pregnancy surgery treating endometriosis. The altered inflammatory and immune environment in endometriosis may be a plausible biological factor contributing to poorer pregnancy outcomes. However, a clear common pathophysiology is yet to be elucidated. Despite an initial database search generating 824 abstracts for possible inclusion, only three studies were included. Two studies were retrospective cohort studies, and one was a case-control study and were all at risk of critical bias due to a lack of describing data handling. From the authors' assessment, they were unable to reliably conclude on the association of pre-pregnancy endometriosis surgery and adverse obstetric outcomes. The authors noted the importance of well-designed prospective studies to confirm or refute possible associations between endometriosis and obstetric complications, and how either medical or surgical management alters this risk. Until such studies are carried out, pathophysiology or the extent of effect of surgical intervention pre-pregnancy remains inconclusive.

In the setting of endometriosis, it is crucial to identify and validate the risks and implications of associated comorbid conditions, to help reduce diagnostic delay and to improve the quality of life of people with endometriosis. In this Research Topic, a wide range of comorbidities were assessed, revealing possible genetic and clinical links between endometriosis and other gynecological and non-gynecological comorbidities. To enhance our understanding of endometriosis and to achieve the ultimate aim of improving endometriosis patients quality of life, more research and clinical attention to the association between endometriosis and other diseases is warranted.

## Author Contributions

All authors listed have made a substantial, direct, and intellectual contribution to the work and approved it for publication.

## Conflict of Interest

The authors declare that the research was conducted in the absence of any commercial or financial relationships that could be construed as a potential conflict of interest.

## Publisher's Note

All claims expressed in this article are solely those of the authors and do not necessarily represent those of their affiliated organizations, or those of the publisher, the editors and the reviewers. Any product that may be evaluated in this article, or claim that may be made by its manufacturer, is not guaranteed or endorsed by the publisher.

